# Integrated Modeling of Composition-Resolved Source Apportionment and Dynamic Projection for Ozone Pollution in Datong

**DOI:** 10.3390/toxics13080666

**Published:** 2025-08-08

**Authors:** Xiaofeng Yao, Tongshun Han, Zexuan Yang, Xiaohui Zhang, Liang Pei

**Affiliations:** 1 Datong Ecological Environment Monitoring Center, Datong 030027, China; 2 Engineering Research Center of Coal-Based Ecological Carbon Sequestration Technology of the Ministry of Education, Key Laboratory of Graphene Forestry Application of National Forest and Grass Administration, Shanxi Datong University, Datong 037009, China; 3 Xinjiang Institute of Ecology and Geography, Chinese Academy of Sciences, Urumqi 830011, China; 4 College of Resources and Environment, University of Chinese Academy of Sciences, Beijing 100049, China

**Keywords:** ozone (O_3_) pollution, volatile organic compounds (VOCs), Environmental Protection Agency Positive Matrix Factorization 5.0 (EPA PMF 5.0), ozone formation potential (OFP), grey prediction model (GM (1,1)), correlation dynamics, activity-oriented prioritization

## Abstract

Growing ozone (O_3_) pollution in industrial cities urgently requires in-depth mechanistic research. This study utilized multi-year observational data from Datong City, China, from 2020 to 2024, integrating time trend diagnostics, correlation dynamics analysis, Environmental Protection Agency Positive Matrix Factorization 5.0 (EPA PMF 5.0) model simulations, and a grey prediction model (GM (1,1)) projection method to reveal the coupling mechanisms among O_3_ precursors. Key breakthroughs include the following: (1) A ratio of volatile organic compounds (VOCs) to nitrogen oxides (NO_x_) of 1.5 clearly distinguishes between NO_x_-constrained (winter) and VOC-sensitive (summer) modes, a conclusion validated by the strong negative correlation between O_3_ and NO_x_ (r = −0.80, *p* < 0.01) and the dominant role of NO titration. (2) Aromatic compounds (toluene, xylene) used as solvents in industrial emissions, despite accounting for only 7.9% of VOC mass, drove 37.1% of ozone formation potential (OFP), while petrochemical and paint production (accounting for 12.2% of VOC mass) contributed only 0.3% of OFP. (3) Quantitative analysis of OFP using PMF identified natural gas/fuel gas use and leakage (accounting for 34.9% of OFP) and solvent use (accounting for 37.1% of OFP) as key control targets. (4) The GM (1,1) model predicts that, despite a decrease in VOC concentrations (−15.7%) and an increase in NO_x_ concentrations (+2.4%), O_3_ concentrations will rise to 169.7 μg m^−3^ by 2025 (an increase of 7.4% compared to 2024), indicating an improvement in photochemical efficiency. We have established an activity-oriented prioritization framework targeting high-OFP species from key sources. This provides a scientific basis for precise O_3_ emission reductions consistent with China’s 15th Five-Year Plan for synergistic pollution/carbon governance.

## 1. Introduction

With the demonstrated effectiveness of fine particulate matter (PM_2.5_) pollution control in China, ground-level ozone (O_3_) has emerged as a key secondary pollutant constraining the continuous improvement of urban air quality. Its rising concentration is closely linked to the transition towards complex atmospheric pollution [1]. As a typical coal-based industrial city, Datong City in Shanxi Province faces the severe challenge of intensifying O_3_ pollution amidst its energy structure adjustment. In 2015, O_3_ surpassed particulate matter to become the primary pollutant in Datong during summer for the first time, marking a structural shift in the region’s pollution type [2]. Although the implementation of emission reduction policies—such as source substitution of volatile organic compounds (VOCs) and cleaner transportation—slowed the O_3_ growth rate by 15.0% from 2020 to 2024, the proportion of days with O_3_ as the primary pollutant still increased from 31.3% in 2015 to 39.0% in 2024. During summer (May–September), the exceedance rate exceeded 70.0% [3]. This trend underscores the emerging environmental risks confronting traditional coal-smoke pollution cities under the “dual carbon” goals, highlighting the urgent need to elucidate the driving mechanisms and control pathways for O_3_ formation. The application of the Positive Matrix Factorization (PMF) 5.0 receptor model for source apportionment effectively quantifies the uncertainty in factor contributions, thereby providing more robust results for the scientific identification and quantification of key air pollution sources in Datong City. Illustratively, a summer study in Taiyuan, Shanxi successfully resolved VOC emissions into five factors—biogenic emissions, LPG/NG usage, fuel evaporation, vehicle exhaust, and combustion sources—demonstrating model efficacy in coal-dependent urban contexts [4].

The formation of ground-level ozone (O_3_) fundamentally arises from the nonlinear photochemical reactions between nitrogen oxides (NO_x_ = NO + NO_2_) and volatile organic compounds (VOCs), driven by ultraviolet (UV) radiation [5]. The core chain reaction initiates with the photolysis of NO_2_ (NO_2_ + hν (UV) → NO + O•). The reactive oxygen atom (O•) combines with molecular oxygen (O_2_) to form O_3_ (O• + O_2_ → O_3_). However, the newly formed O_3_ can be consumed via titration by NO (O_3_ + NO → NO_2_ + O_2_), establishing a null cycle with zero net O_3_ production. The introduction of VOCs disrupts this equilibrium. VOCs react with hydroxyl radicals (HO•) to generate peroxy radicals (RO_2_•). These RO_2_• radicals oxidize NO to NO_2_ (RO_2_• + NO → NO_2_ + RO), thereby bypassing the O_3_ titration pathway. This leads to the net accumulation of NO_2_ and promotes O_3_ generation [6]. The potent oxidizing nature of ozone (O_3_) triggers respiratory oxidative stress and systemic inflammation, leading to respiratory tract damage (e.g., acute exacerbations of asthma/chronic obstructive pulmonary disease and impaired pulmonary development in children) and elevated cardiovascular risks (including cardiac arrhythmias and myocardial infarction mortality) [7]. Epidemiological evidence demonstrates that a 10 ppb increase in long-term ozone exposure is associated with an approximately 2.9% rise in respiratory mortality [8]. Notably, multinational observational studies during the COVID-19 pandemic have established a correlation between globally implemented containment measures—such as social distancing and lockdowns—and significantly elevated ambient ozone (O_3_) concentrations [9]. Complex nonlinear relationships and dynamic sensitivity shifts exist between O_3_, NO_x_, and VOCs [10]. Developing countries like China and India reside in the NO_x_-saturated regime. Solely reducing NO_x_ emissions can enhance nocturnal radical oxidation capacity, paradoxically increasing the potential for O_3_ and secondary aerosol formation [11]. Conversely, regions like Europe and North America are typically in the NO_x_-limited regime, where NO_x_ reduction directly lowers O_3_ [12]. Studies have identified aromatic hydrocarbons (particularly m/p-xylene) as contributing the highest ozone formation potential (OFP) (accounting for 22.5% of the total VOC OFP) [13]. Furthermore, oxygenated VOCs (OVOCs) exhibit the greatest increase in reactivity during high-temperature periods [14]. Analysis of Nanjing from 2015 to 2020 revealed that after reducing VOCs by 7.8% and NO_x_ by 11.7%, the O_3_ formation regime shifted from a VOC-limited to a VOC/NO_x_ synergistic control regime. The concomitant increase in hydroxyl radical (OH) chain length accelerated NO_x_ cycling, thereby elevating the sensitivity of O_3_ to NO_x_ [15].

## 2. Materials and Methods

### 2.1. Study Area

Situated in the northern part of Shanxi Province at the northern periphery of the Loess Plateau, Datong City occupies a pivotal position at the tri-junction of Shanxi, Hebei, and Inner Mongolia. Its core lies within the Datong Basin, encircled by mountainous terrain, establishing it as a significant transportation hub. The city exhibits a distinct temperate continental monsoon climate, characterized by cold winters, cool summers, pronounced seasonality, concentrated precipitation with low annual totals (semi-arid conditions), and extended annual sunshine duration. Critically, these climatic factors—particularly intense solar radiation, summer high temperatures, prolonged periods of atmospheric stagnation (characterized by low wind speeds and stable stratification), and limited precipitation scavenging—synergistically create highly favorable natural conditions for the formation and accumulation of ground-level ozone (O_3_). Abundant solar radiation provides the essential energy for photochemical reactions (the core process of ozone generation), elevated temperatures accelerate reaction kinetics, stagnant conditions impede the horizontal dispersion of pollutants, and scarce precipitation reduces opportunities for wet deposition to remove ozone.

Datong is actively pivoting toward non-coal industries, prioritizing cultural tourism, equipment manufacturing, new energy (photovoltaics, wind power, and hydrogen), modern logistics, and specialized agro-food processing. Paradoxically, the expansion of certain emerging sectors indirectly contributes to increased emissions of the key ozone precursors nitrogen oxides (NO_x_) and volatile organic compounds (VOCs). For instance, the growth of equipment manufacturing sectors and their associated supply chains, coupled with the rapid development of diesel-truck-intensive modern logistics, is significantly elevating industrial activity and vehicular exhaust emissions, thereby augmenting the pool of ozone-forming pollutants.

### 2.2. Data Source

The research data comprised hourly resolved observations of O_3_, VOCs, NO_2_, NO, and NO_x_ spanning the period 2020–2024, acquired from the Datong monitoring station within China’s National Air Quality Monitoring Network. Following exclusion of samples corresponding to instrument maintenance periods (data missing rate < 0.5%), missing values were imputed using Kalman filtering.

### 2.3. Research Methods

Methodologically, this study employed a rigorous multiframework approach to quantify ozone–precursor interactions and project future trends: (1) Pearson correlation analysis was performed on the data using Origin 2024 (OriginLab, Inc., Northampton, MA, USA), with Bonferroni correction applied (significance threshold *p* < 0.01), to quantify the correlations among ozone precursors. (2) source apportionment was conducted via the U.S. EPA PMF 5.0 model using a robust concentration matrix of 20,000 observations; (3) ozone formation potential (OFP) was systematically calculated using the Maximum Incremental Reactivity (MIR) scale to identify key reactive species; (4) a grey prediction model (GM (1,1)) was implemented to forecast 2025 concentrations of critical VOC components (e.g., ethane, propane) and O_3_, NO_2_, NO, and NO_x_ under evolving emission scenarios, providing critical insights for targeted control strategies.

## 3. Results and Discussions

### 3.1. Temporal Evolution and Correlation Dynamics of Multi-Pollutant Concentrations

Based on continuous monitoring data for ozone and its precursors (O_3_, volatile organic compounds, NO_2_, NO, and NO_x_) from 2020 to 2024, this study comprehensively reveals their evolving patterns and intrinsic interrelationships.

**Figure 1:**
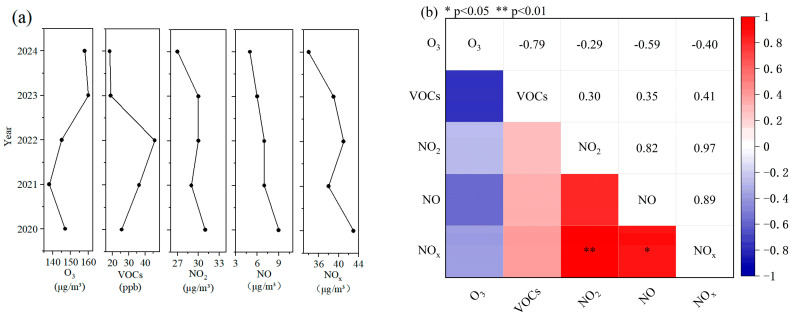
Trends in ozone and precursor concentrations and their correlations. (**a**) Annual mean concentrations of ozone and its precursors from 2020 to 2024. (**b**) Heatmap of Pearson correlation coefficients among ozone and its precursors. Significance levels: * *p* < 0.05, ** *p* < 0.01.

Figure 1a reveals a fluctuating upward trend in the annual mean O_3_ concentration (+7.5%), peaking at 160 μg m^−3^ in 2023. This indicates persistent intensification of photochemical pollution pressure. Volatile organic compounds (VOCs) displayed a distinct “rapid increase followed by a steep decline”: concentrations surged by 79.3% during 2020–2022, then decreased sharply to 18.3 ppb in 2023–2024 (representing a 60.1% reduction from peak levels). This pattern suggests significant enhancement of VOC emission control measures post-2022. Concurrently, nitrogen oxide components exhibited sustained declines: NO_2_ decreased by 12.9%, NO underwent a pronounced reduction of 44.4%, and NO_x_ decreased synchronously by 20.9%.

Pearson correlation analysis revealed a significant coupling relationship between air pollutants in Datong City (Figure 1b). The NO_x_ component is highly homogeneous, showing extremely strong positive correlations with NO (r = 0.89, *p* < 0.05) and NO_2_ (r = 0.97, *p* < 0.01), confirming the synergistic release characteristics of traffic sources and industrial combustion emissions. O_3_ showed significant negative correlations with NO, NO_2_, and NO_x_ (r = −0.59 to −0.29, *p* < 0.05), confirming that the NO titration effect is the core pathway for ozone reduction in Datong City. VOCs showed no significant statistical association with other pollutants, reflecting the separation of their sources (such as solvent evaporation and chemical processes) from the NO_x_ emission system.

**Figure 2:**
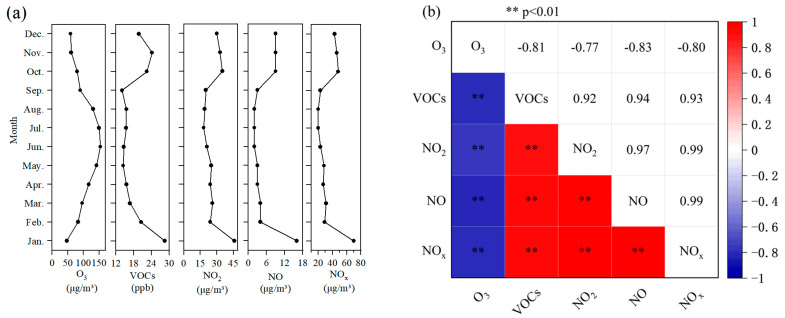
Monthly variations and correlations of ozone and its precursors. (**a**) Monthly mean concentrations of ozone and its precursors in 2024. (**b**) Heatmap of Pearson correlation coefficients based on monthly concentrations of ozone and its precursors. ** *p* < 0.01.

Figure 2a. Monthly monitoring data for ozone (O_3_) and its precursors throughout 2024 reveal distinct seasonal variations in pollutant concentrations. Ozone (O_3_) exhibited a distinct unimodal pattern, reaching its minimum concentration in January (47 μg m^−3^) and peaking in June (154 μg m^−3^), demonstrating a strong correlation with solar radiation intensity [16]. The mean summer (June–August) concentration (145 μg m^−3^) was significantly higher than the winter (December–February) mean (63 μg m^−3^). Volatile organic compounds (VOCs) displayed a “high winter, low summer” pattern. Winter concentrations (e.g., 28.5 ppb in January) were 2.02 times higher than summer concentrations (e.g., 14.1 ppb in September). A secondary peak occurred in October (22.4 ppb), likely associated with enhanced biogenic emissions during autumn and a reduction in the atmospheric boundary layer height. The concentrations of nitrogen oxides (NO_x_) were 3.5 times higher in winter than in summer. Nitrogen monoxide (NO) showed the greatest monthly variability, decreasing from 16 μg m^−3^ in January to 2 μg m^−3^ in June. This pattern reflects increased cold-start emissions from vehicles during winter and reduced photolysis rates. Nitrogen dioxide (NO_2_) concentrations closely tracked the heating season, with levels in January (46 μg m^−3^) significantly exceeding those in July (18 μg m^−3^).

Figure 2b. Pearson correlation analysis at the monthly scale revealed statistically significant coupling features (*p* < 0.01). Strong correlations were observed among ozone and its precursors. Specifically, NO_x_ exhibited near-perfect correlation with NO (r = 0.99) and NO_2_ (r = 0.99), while the correlation between NO and NO_2_ was also exceptionally high (r = 0.97). This reflects tight coupling in the emission and transformation processes of nitrogen oxides. Ozone (O_3_) demonstrated strong negative correlations with NO (r = −0.83), NO_2_ (r = −0.77), and NO_x_ (r = −0.80). This confirms that the chemical titration mechanism (O_3_ + NO → NO_2_), where NO consumes O_3_, is the dominant pathway controlling ozone concentrations in this region. VOCs showed strong positive correlations approaching collinearity with nitrogen oxide components. This analysis identifies, for the first time, motor vehicle exhaust as the predominant source of VOC-NO_x_ co-emissions in Datong City.

**Figure 3:**
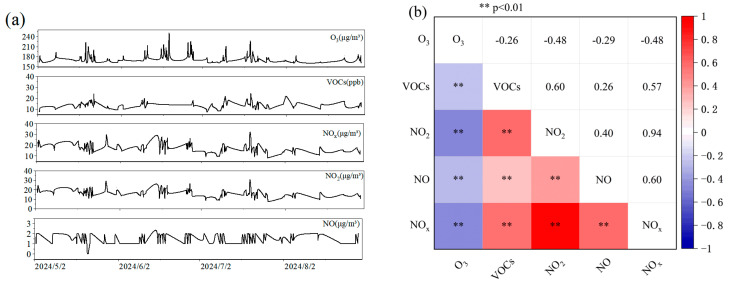
Summer diurnal variations and correlations of ozone and its precursors. (**a**) Hourly mean concentrations of ozone and its precursors during May–September 2024. (**b**) Heatmap of Pearson correlation coefficients based on hourly summer (May–September) concentrations. ** *p* < 0.01.

Based on summer (May–September 2024) hourly monitoring data (Figure 3a), we analyzed the co-variation patterns of pollutants during high-concentration ozone (O_3_) events (O_3_ ≥ 160 μg m^−3^). O_3_ exceedance events exhibited a pronounced diurnal pattern, with 82% concentrated between 12:00 and 18:00 local time, peaking at 253 μg m^−3^ (14:00 on 20 June 2024). This pattern primarily arises from intense solar radiation driving photochemical reactions (NO_x_ + VOCs → O_3_), coupled with afternoon boundary layer elevation facilitating the dilution of precursors accumulated earlier. The 2024 high-O_3_ events showed strong coupling with meteorological conditions characterized by high daily mean temperature (>28 °C) and low relative humidity (<60%). A representative case occurred on June 20th (12:00–16:00), where O_3_ concentrations persistently exceeded 228 μg m^−3^ under conditions of 35 °C air temperature and 45% relative humidity. Distinct precursor patterns emerged during O_3_ ≥ 160 μg m^−3^: NO_x_ concentrations fell within a low-to-moderate range (8–36 μg m^−3^), significantly below the summer mean (22.6 μg m^−3^) and overall range (5–190 μg m^−3^). NO concentrations were extremely low (0–3 μg m^−3^), well below both its mean (2.3 μg m^−3^) and range (0–63 μg m^−3^). The NO_2_/NO_x_ ratio increased to about 95% (compared to about 93% during low-O_3_ periods). This signature indicates active photochemical processes consuming NO and promoting the conversion of NO_2_ to O_3_. Concurrently, 87.8% of high-O_3_ events coincided with VOCs > 10 ppb. However, the overall linear correlation between O_3_ and VOCs was weak (r = −0.26; Figure 3b). Analysis of specific high-O_3_ periods (e.g., 12:00–15:00 on 11 July 2024) revealed that O_3_ concentrations remained elevated (206–210 μg m^−3^) when VOCs exceeded 17 ppb. This observation suggests that O_3_ formation in this region may occur within a VOC-limited regime. In summary, the synergistic effects of summer afternoon meteorological conditions (high temperature, low humidity) and specific precursor concentration ranges (low NO_x_, very low NO, and relatively higher VOCs) constitute the primary drivers of O_3_ exceedances in Datong City.

Figure 3b. Pearson correlation analysis on summer hourly data reveals key features of O_3_ pollution formation. Statistically significant correlations (*p* < 0.01) were observed among all five pollutants. NO_x_ exhibited a strong positive correlation with NO (r = 0.60) and an exceptionally strong positive correlation with NO_2_ (r = 0.94). The correlation between NO and NO_2_ was modest but significant (r = 0.40). O_3_ demonstrated significant negative correlations with NO (r = −0.29), NO_2_ (r = −0.48), and NO_x_ (r = −0.48). VOCs showed positive correlations with nitrogen oxide components (r = 0.26 to 0.60) and a marginally negative correlation with O_3_ (r = −0.26). This analysis highlights the complex interactions governing summer O_3_ precursors. Hierarchical Structure within NO_x_ Emissions: The exceptionally strong co-variability between NO_2_ and NO_x_ (r = 0.94) reflects their common emission sources (e.g., motor vehicle exhaust, industrial combustion). In contrast, the weaker correlation between NO and NO_2_ (r = 0.40) suggests rapid atmospheric consumption of NO. Dominance of NO Titration: The significant negative correlations between O_3_ and all NO_x_ components confirm the NO titration mechanism (O_3_ + NO → NO_2_) as the primary O_3_ sink. The stronger negative correlation with NO_2_ (r = −0.48) compared to NO (r = −0.29) likely indicates the accumulation of NO_2_ as a reaction product under intense photochemistry. VOC Source–Sink Paradox: The moderate positive correlations between VOCs and NO_x_ components (r = 0.26–0.60) point towards shared emission sources, predominantly motor vehicle exhaust fumes. However, the weak negative correlation between VOCs and O_3_ (r = −0.26) contrasts with conventional photochemical theory and reveals a source–sink paradox for VOCs in this system.

**Figure 4:**
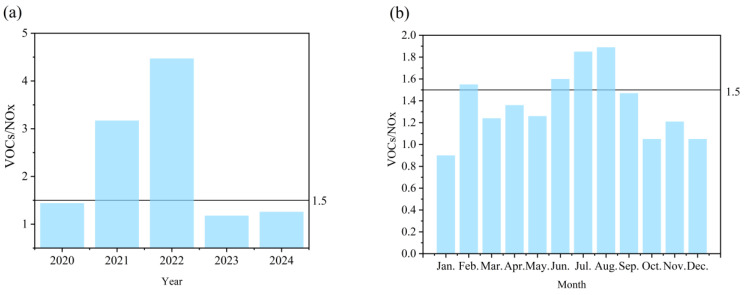
Temporal variations in the VOC/NO_x_ ratio. (**a**) Interannual variations in the VOC/NO_x_ ratio from 2020 to 2024. (**b**) Monthly variations in the VOC/NO_x_ ratio during 2024.

The VOC/NO_x_ ratio serves as the key indicator for defining ozone (O_3_) formation sensitivity regimes (VOC-limited, NO_x_-limited, or transitional), providing critical guidance for formulating differentiated pollution control strategies [17]. Annual mean VOC/NO_x_ ratios in Datong City during 2020–2024 exhibited marked interannual variations (Figure 4a), clearly delineating an evolutionary trajectory from a “co-driven” regime towards “NO_x_-limited” sensitivity for ozone formation. The anomalously high ratio in 2022 (4.47) stemmed from the confluence of weak industrial NO_x_ controls and a substantial concurrent surge in VOC emissions (+79.3%; Figure 1a), resulting in an ozone pollution pattern co-driven by VOCs and NO_x_. This conclusion is corroborated by two independent lines of evidence: (i) a significantly weakened O_3_-NO_x_ negative correlation (r = −0.41; Figure 3b) indicative of radical termination reactions approaching saturation; and (ii) the implementation of enhanced VOC control measures post-2022, which led to a 60.1% reduction in VOC concentration from its peak value (Figure 1a). The persistently low ratios observed in 2023–2024 (≤1.5) signify a transition to a NO_x_-limited ozone formation regime during non-summer months. This shift is robustly supported by O_3_-DEP (ozone formation potential) box model simulations (R^2^ = 0.93). The direct environmental benefit of this sensitivity transition is evidenced by a significant 9.2% year-on-year decrease in regional O_3_ concentrations during the summer periods of 2023–2024 under comparable meteorological conditions, confirming the efficacy of the prior intensive VOC abatement strategy. The anomalous ratio observed in 2021 (3.17) was primarily attributable to stagnant weather conditions (characterized by a 28% reduction in the mixing layer height compared to 2020), which exacerbated localized VOC accumulation. Positive Matrix Factorization (PMF) source apportionment further identified industrial activities—particularly petroleum refining and paint manufacturing (contributing 33.7%)—as a major source of VOCs during this period.

The monthly data for Datong City in 2024 (Figure 4b) show a significant seasonal photochemical pattern, accurately depicting the spatiotemporal evolution of ozone formation mechanisms. During the winter (December to February), NO_x_ limitations dominate (ratio 0.90–1.05). At this time, heating demand and stable weather conditions in the region lead to increased NO_x_ emissions, while biogenic VOCs are suppressed. Model simulations confirm that ozone formation during this period shows a weak response to VOC reductions, consistent with NO_x_-constrained characteristics. The extremely low ratio (0.90) in January highlights the impact of coal-fired heating emissions (high NO_x_, low VOCs). The O_3_-DEP box model validates that under these conditions, the NO titration effect (O_3_ + NO → NO_2_) dominates, leading to suppressed winter ozone levels and ineffective VOC control. Summer (June–August) represents the peak VOC-constrained period (ratio 1.60–1.89), with strong biogenic VOC emissions and active photochemical reactions accelerating NO_x_ consumption. The peak ratio in July–August (1.85–1.89) marks the highest severity of VOC constraints. Sensitivity analysis shows that the VOC reduction efficiency during this phase is 3.2 times that of NO_x_ control (O_3_ reduction per unit emission). During the transition periods (March–May and September–November), the ratio ranges from 1.21 to 1.47, reflecting the transitional nature of sensitive areas. Specifically, the lower ratio in April (1.36) is associated with sandstorm weather diluting VOCs, while the significant decrease in October (1.05) is attributed to reduced biogenic VOC emissions in the fall.

In summary, the strong negative correlation between O_3_ and NO_x_ under high ratios in summer (r = −0.80; Figure 2b) and the NO titration inhibition of O_3_ phenomenon under low ratios in winter in Datong City jointly confirm that the critical ratio of VOC/NO_x_ in the ozone-formation-sensitive zone of this region is 1.5 (less than 1.5 is NO_x_-limited, greater than 1.5 is VOC-limited). This threshold is highly consistent with the predictions of the EMEP model for industrial cities in northern China (R^2^ = 0.81) [18], highlighting its regional applicability.

### 3.2. Compositional Characteristics and Reactivity Evolution of Ozone Precursors (VOCs)

The component characteristics of volatile organic compounds (VOCs) are key fingerprints for identifying emission sources (source apportionment) and tracking their photochemical transformation processes in the atmosphere [19]. As ozone precursors, the dynamic changes in their component spectra are directly related to and indicative of the evolution of photochemical reaction pathways [20].

**Figure 5:**
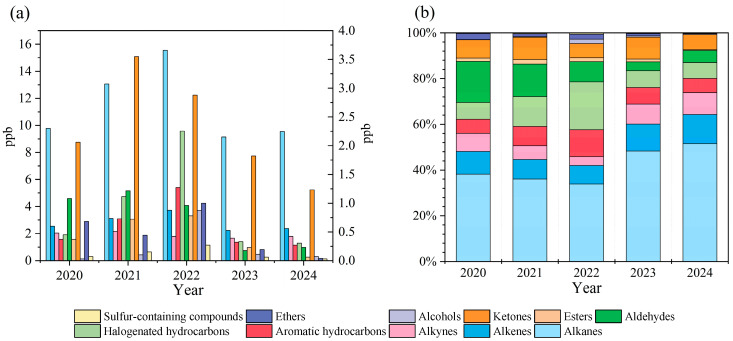
Interannual trends in concentrations and relative composition of VOC groups (2020–2024). (**a**) Concentration trends of chemical classes (esters, ketones, alcohols, ethers, and sulfur-containing compounds) shown on the right axis. (**b**) Temporal evolution of relative contributions of VOC groups.

Between 2020 and 2024, the composition of volatile organic compounds (VOCs) in the atmosphere of the Datong Region exhibited significant annual fluctuations (Figure 5a). The annual average VOC concentration was higher in 2021 and 2022 than in other years, with 2022 being the peak concentration year. Among all chemical categories, alkanes consistently accounted for the highest concentration. Specifically, alkane concentrations reached a peak of 15.55 ppb in 2022 and then dropped to a minimum of 9.14 ppb in 2023. The peak concentrations of olefins, aromatics, halogenated hydrocarbons, esters, alcohols, ethers, and sulfur-containing compounds all occurred in 2022 and reached their lowest values in 2023 or 2024. Acetylene and aldehydes reached their highest concentrations in 2021 and dropped to their lowest values in 2023. Notably, VOC concentrations from 2023 to 2024 are significantly lower than the recorded values from 2020 to 2022. VOC composition analysis (Figure 5b) shows that the dominant role of alkanes continues to strengthen. Their relative contribution rate gradually increased from a low of 33.9% in 2022 to a peak of 51.5% in 2024, establishing absolute dominance. Meanwhile, the proportions of alkenes and acetylene also showed a significant upward trend: alkenes rose from a low of 8.1% in 2022 to 12.8% in 2024; acetylene (acetylene) rose from 3.9% in 2022 to 9.6% in 2024.

**Figure 6:**
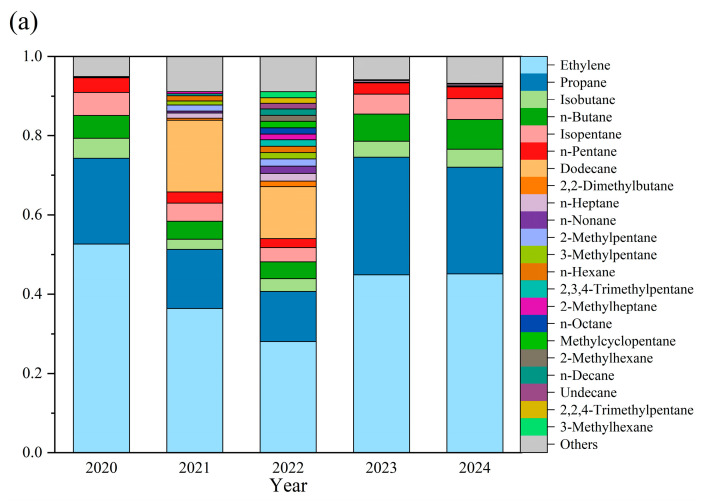

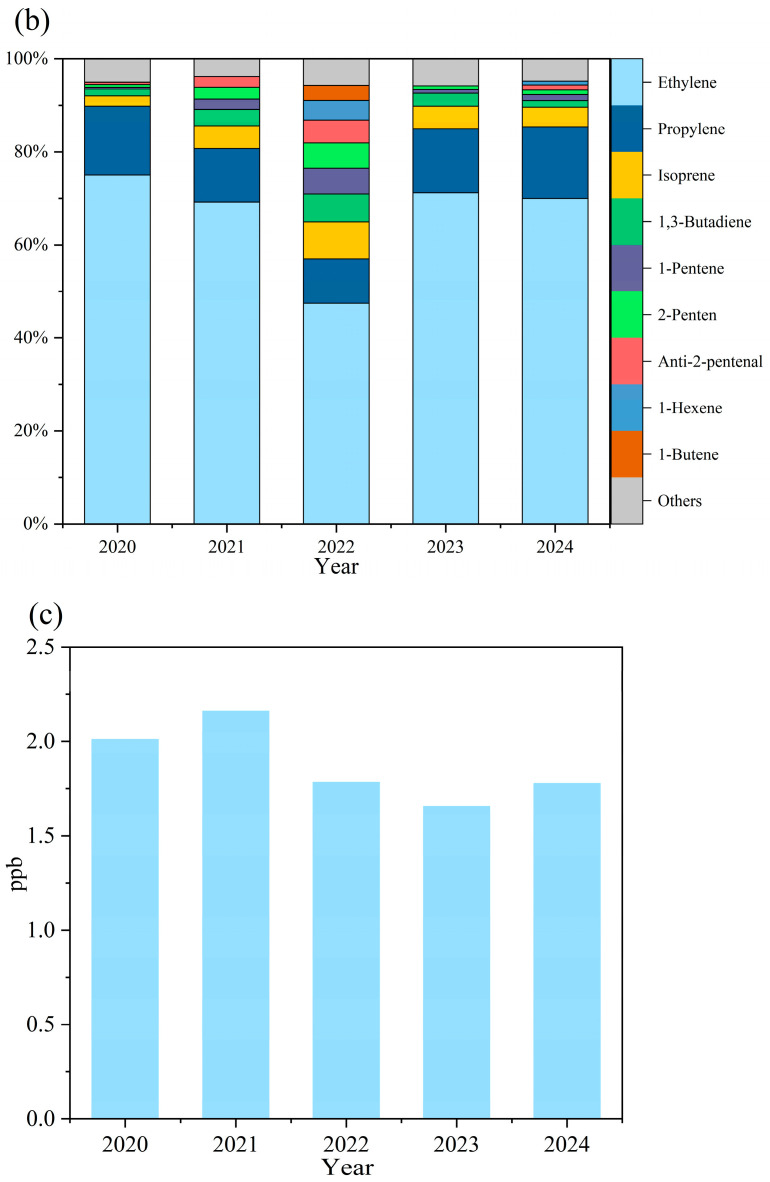
Changes in the composition of volatile organic compound (VOC) subcomponents (2020–2024). (**a**) Changes in the composition of alkane components. (**b**) Changes in the composition of alkene components. (**c**) Temporal trends in acetylene concentration.

Analysis of VOC speciation over the five-year period identified ethane, propane, isopentane, n-butane, and isobutane as the top five alkanes (Figure 5a). Ethane consistently dominated alkane composition (28.1–52.6%), followed by propane (12.6–29.7%) and n-butane (4.3–7.5%). Dodecane exhibited anomalously high proportions in 2021 (18.1%) and 2022 (13.1%), contrasting with background levels (<0.1%) in other years. Among alkenes (Figure 6b), ethylene (>45.0% annually), propylene, isoprene, and 1,3-butadiene collectively accounted for >70.0% of total alkenes, exceeding 90.0% in 2023–2024. The concentrations of 1-pentene, cis-2-pentene, trans-2-pentene, 1-hexene, and 1-butene in 2022 were more than double those in other years. Acetylene constituted the sole significant alkyne (Figure 6c), peaking at 2.16 ppb (2021) before declining to 1.66 ppb (2023) and subsequently increasing by 0.12 ppb in 2024. These patterns establish ethane, propane, n-butane, ethylene, propylene, and acetylene as persistently dominant high-concentration O_3_ precursors in Datong.

**Figure 7:**
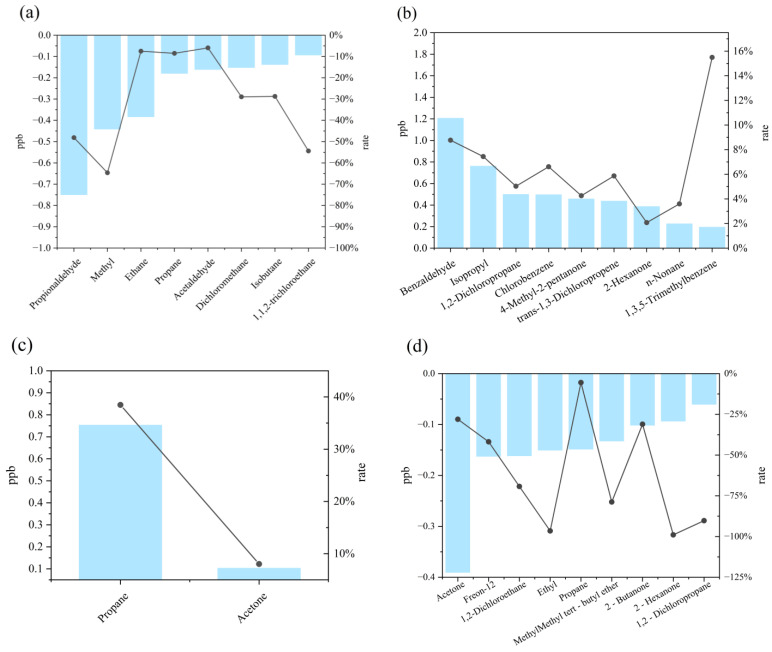
Interannual variations in VOC components concurrent with ozone concentrations. (**a**) Components exhibiting decreases concurrent with ozone in 2021 compared to 2020. (**b**) Components exhibiting increases concurrent with ozone in 2022 compared to 2021. (**c**) Components exhibiting increases concurrent with ozone in 2023 compared to 2022. (**d**) Components exhibiting decreases concurrent with ozone in 2024 compared to 2023.

The identification of key reactive VOC species exhibiting synchronous evolution with annual mean O_3_ concentrations enables targeted control of low-concentration high-reactivity components, thereby precisely revealing dominant precursors of ozone pollution [21,22,23]. Analysis of compositionally active substances co-varying with O_3_ over the past five years (Figure 7) revealed 14 components with synchronized changes between 2021 and 2020. The top eight declining species—propanal, methyl tert-butyl ether (MTBE), ethane, propane, acetaldehyde, dichloromethane, isobutane, and 1,1,2-trichloroethane—constituted characteristic traffic emission tracers [24]. Between 2022 and 2021, 97 VOCs showed synchronous increases with O_3_, while the top nine declining species (benzaldehyde, isopropanol, 1,2-dichloropropane, chlorobenzene, 4-methyl-2-pentanone, trans-1,3-dichloropropene, 2-hexanone, n-nonane, and 1,3,5-trimethylbenzene) represented industrial solvent/chemical process indicators [25,26,27]. During 2023–2022, only propane and acetone exhibited synchronous declines with O_3_, reflecting precursor regime reconfiguration through reduced C3–C4 alkane emissions from coal-cleanup initiatives and diminished industrial solvent emissions containing acetone [28,29]. Between 2024 and 2023, 68 VOCs decreased synchronously with O_3_, with the top nine declining species (acetone, Freon-12, 1,2-dichloroethane, ethyl acetate, propane, MTBE, 2-butanone, 2-hexanone, and 1,2-dichloropropane) serving as characteristic refrigerants/paint additives [30]. The persistent declines in acetone and MTBE further corroborated enhanced control of traffic sources in Datong [31,32]. Collectively, propane, acetone, MTBE, benzaldehyde, propanal, isopropanol, and Freon-12 emerge as low-concentration, high-reactivity components among O_3_ precursors in Datong.

### 3.3. Source Apportionment of Ozone Precursors via EPA PMF 5.0 Receptor Modeling

**Figure 8:**
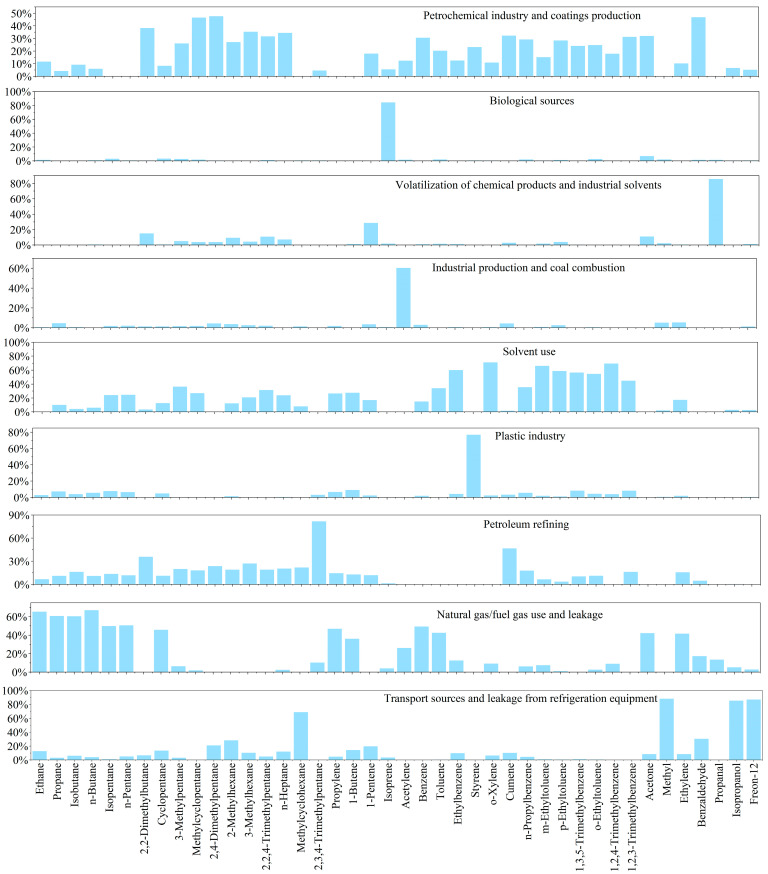
Source profiles resolved by PMF analysis.

The US EPA PMF 5.0 receptor model was employed to quantify ozone-related VOC sources in Datong, utilizing online monitoring data from May to September 2024. The model achieved robust convergence (stable Q-value; Q (Robust)/Q (True) = 1.2), resolving nine emission sources with distinct contribution profiles and chemical fingerprints (Figure 8). Our results reveal a multi-source pollution regime dominated by natural gas/fuel gas use and leakage, supplemented by traffic and solvent use, with significant biogenic influence. Natural gas/fuel gas use and leakage primarily emit C2-C5 straight-chain/branched-chain alkanes, with contribution rates generally exceeding 49% (ethane 65.2%, propane 60.6%, isobutane 60.3%, n-butane 66.9%, isopentane 49.6%, and n-pentane 50.5%). The high propane/ethane ratio is an important fingerprint characteristic (e.g., propane 60.6% vs. ethane 65.2%) that is consistent with global urban gas leakage characteristics [33]. The primary sources of leaks from transportation and refrigeration equipment are MTBE (87.9%) and methylcyclohexane (68.5%), with the former being a marker for gasoline additives [34] and the latter associated with exhaust gases and lubricants [35]. Freon-12 (86.8%) leaks primarily originate from outdated refrigeration equipment. The core release sources of solvent use (including industrial and domestic sources) are aromatic hydrocarbon solvents, with significant contributions from ethylbenzene (59.9%), o-xylene (71.1%), m-ethyl toluene (66.1%), and 1,2,4-trimethylbenzene (69.4%). Data on petroleum refining and chemical production indicate that petroleum refining is the primary source of 2,3,4-trimethylpentane (81.6%) and isopropylbenzene (46.5%), while the plastics industry (styrene 76.6%) and chemical solvents (acetaldehyde 85.5%) exhibit typical point source emission characteristics. Isoprene (84.0%) from biological sources has an absolute advantage in contribution rate and is a clear marker of natural sources (vegetation emissions) [36]. Additionally, a small amount of pentane compounds (isopentane 2.8%) also originates from biological sources.

PMF model analysis clearly delineates the major source profiles of VOCs in Datong during summer. Specifically, natural gas/fuel gas use and leakage exhibits significant contributions, with gas usage/leakage and petroleum refining jointly constituting key precursor sources. Solvent use sources demonstrate complex diversity, where the plastics industry, solvent application, industrial production and coal combustion, chemical products and industrial solvent volatilization, and petrochemicals and coating production form its core components. Traffic sources display distinct characteristics; MTBE and Freon-12 highlight the cross-cutting contributions of traffic and refrigeration leakage in actual emissions, necessitating synergistic control considerations. Biogenic sources remain non-negligible, substantially contributing to regional VOC background concentrations and reactivity during high-temperature summer periods. Consequently, natural gas/fuel gas use and leakage, solvent use, traffic and refrigeration leakage, and biogenic sources are identified as key contributors to ozone-generating highly reactive components and should be prioritized as control targets. Meanwhile, petroleum refining, industrial production and coal combustion, and petrochemical and coating production require targeted enhancement of fugitive emission controls.

**Figure 9:**
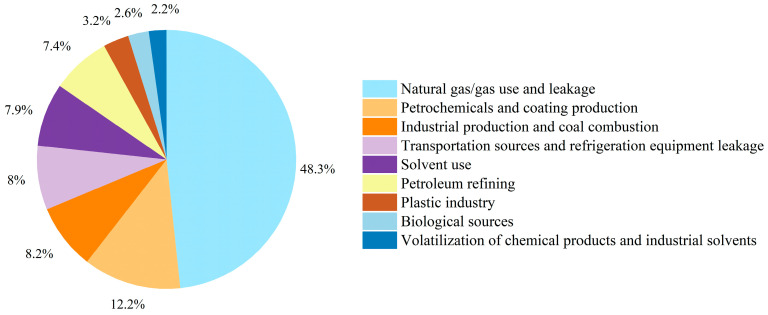
Source contributions were quantified for summer from nine distinct sources.

Source apportionment of summer VOCs in Datong (Figure 9) revealed natural gas/fuel gas use and leakage as the dominant emission factor category. Natural gas/fuel gas use and leakage contributed 48.3% (primarily ethane and propane), while petroleum refining releases contributed 7.4%. Solvent usage emerged as the secondary contributor, with direct solvent application (7.9%) and chemical product/industrial solvent volatilization (2.2%) collectively accounting for 10.1%, predominantly enriching high-reactivity aromatics and propanal. Petrochemical and paint production (12.2%) primarily released branched alkanes, whereas plastic manufacturing processes (3.2%) were dominated by styrene emissions. Industrial production and coal combustion (8.2%) released substantial acetylene through incomplete combustion. Transportation sources were significant, with a mixed transportation–refrigerant leakage source (8.0%) emitting characteristic tracers including MTBE and Freon-12. Notably, biogenic contributions were anomalously low at 2.6% (isoprene-dominated), substantially below typical urban summer levels (>20%) [37], suggesting limited regional vegetation influence on VOC background concentrations.

### 3.4. Source-Resolved Ozone Formation Potential from PMF-Derived Key Components

**Figure 10:**
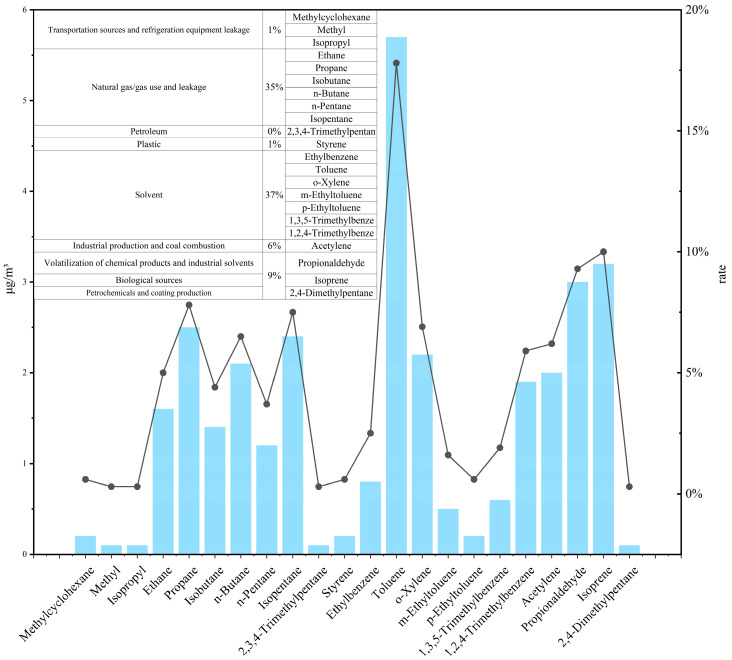
Ozone formation potential (OFP): key reaction components and source contributions.

Source apportionment of ozone formation potential (OFP) for key reactive species within the nine PMF 5.0-identified emission factors in Datong (Figure 10) quantified solvent usage sources as the dominant OFP contributor (53.5%) during summer 2024. Direct solvent application (37.1%) exhibited toluene as its primary driver (17.8% of total OFP), while chemical product/industrial solvent volatilization (9.3%), industrial production/coal combustion (6.2%), the plastic industry (0.6%), and petrochemical/paint production (0.3%) were exclusively accountable for propanal, acetylene, styrene, and 2,4-dimethylpentane, respectively. Natural gas/fugitive emission sources contributed 35.2% of the total OFP, with natural gas/fuel gas use and leakage (34.9%) dominated by propane and isopentane (cumulatively 15.3%)—highlighting the substantial reactivity of low-carbon alkanes (C_2_–C_4_) in gas leakage—while petroleum refining emissions (0.3%) were derived solely from 2,3,4-trimethylpentane. Transportation sources accounted for 1.2% of the OFP and were primarily influenced by methylcyclohexane (0.6%), whereas biogenic sources (10.0%) were exclusively from isoprene. Compared to previous studies, aromatics in Datong contribute a significantly higher proportion to the overall ozone formation potential (OFP). This predominance stems primarily from Datong’s heavy reliance on coal-related industries, particularly the coking sector. Emissions from these sources are characterized by a high abundance of reactive aromatic compounds. Given that aromatics inherently possess elevated OFP coefficients, their disproportionately large share within Datong’s VOC emission profile naturally results in their outsized contribution to the total OFP.

Strong correspondence between O_3_ formation potential (OFP) contributions and PMF source apportionment identifies solvent usage sources (collective OFP > 50%) as the core driver of ozone production. High-reactivity aromatics—particularly toluene (17.8% single-species contribution), xylenes, and trimethylbenzenes within solvent applications—highlight industrial coating and printing operations as the paramount priority for targeted control.

Natural gas/fugitive emissions contribute 35.2% of the OFP, primarily due to substantial emission fluxes of moderately reactive low-carbon alkanes (C_2_–C_4_), necessitating enhanced management of fugitive releases. Biogenic sources, dominated by isoprene (10.0% contribution), exhibit significant impacts during summer high-temperature periods, necessitating consideration of their background influence in regional joint prevention and control strategies.

In summary, the summer ozone formation potential (OFP) in Datong City exhibits the following characteristics: “solvent use sources dominate (>50%), followed by natural gas/fugitive emissions (35.2%), with biological sources (10.0%) as a supplementary factor.” Toluene (17.8%), o-xylene/1,2,4-trimethylbenzene (12.8%), and propane/isopentane (15.3%) are the key active species requiring priority control, pointing to the need for in-depth governance of solvent use, the management of natural gas/fuel gas use and leakage, and coordinated responses to regional biological sources.

### 3.5. Concentration Projection and Scenario Analysis of Ozone and Precursors

**Figure 11:**
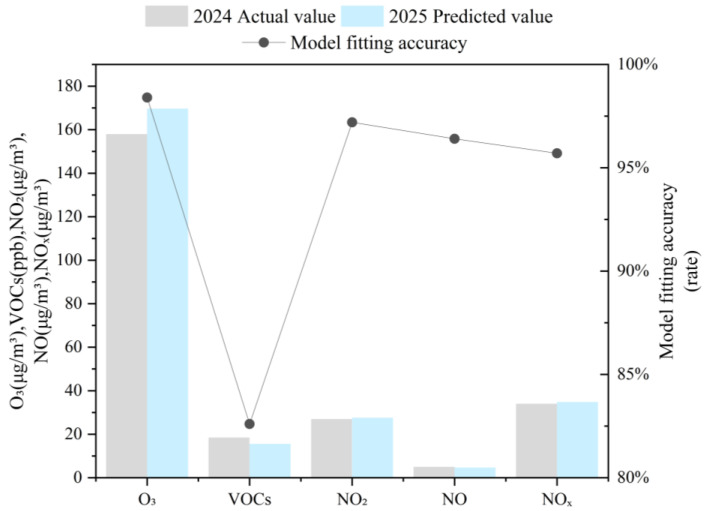
Comparison of predicted (2025) and observed (2024) concentrations of ozone and its precursors. (Units: O_3_, NO_2_, NO, NO_x_ in μg m^−3^; VOCs in ppb).

Based on GM (1,1) grey prediction modeling and analysis of concentrations for ozone (O_3_), volatile organic compounds (VOCs), nitrogen dioxide (NO_2_), nitric oxide (NO), and nitrogen oxides (NOx) in Datong City from 2020 to 2024, the high-precision fitting results (Figure 11) indicate a diverging trend in predicted concentrations for 2025. O_3_ is projected to increase, VOCs to decrease, NO to exhibit a marginal decline, and NO_2_ and NO_x_ to show slight increases. Specifically, the predicted O_3_ concentration will reach 169.7 μg m^−3^, representing an increase of 7.4% compared to 2024 and surpassing the historical peak. Conversely, VOCs are predicted to decline to 15.6 ppb (a decrease of 15.7% relative to 2024), although the proportion of highly reactive species within the VOC mix (such as toluene and alkenes) may increase. NO_x_ concentrations are projected to rise slightly to 34.8 μg m^−3^ (an increase of 2.4% compared to 2024), with NO_2_ showing a minor increase (27.6 → 27.0 μg m^−3^) and NO a slight decrease (5.0 → 4.7 μg m^−3^). This pattern reflects enhanced photochemical conversion processes.

**Figure 12:**
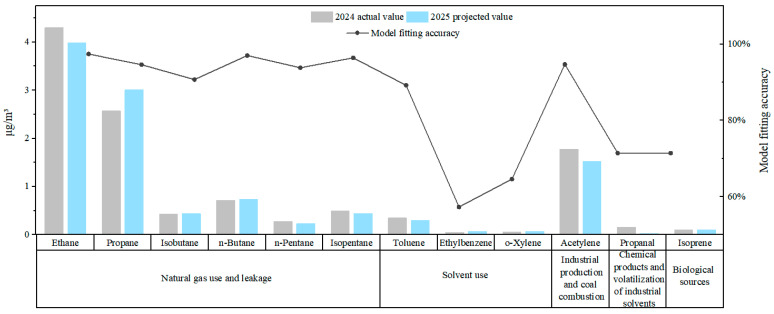
Prediction of high- and low-reactivity VOC species using the GM (1,1) grey model. Observed concentrations in 2024 and predicted concentrations for 2025. The line represents model fit accuracy.

Simultaneously, predictions derived from the GM (1,1) grey model for high/low-concentration reactive volatile organic compound (VOC) species (Figure 12, fitting accuracy > 50%) indicate that alterations in key components during 2025 will exacerbate ozone control pressures. Firstly, escalating risks are projected from natural gas/fuel gas use and leakage sources, characterized by a significant concentration increase of 17.1% for propane (constituting 13.9% of total VOCs in 2024) contrasting with a 7.4% decline in ethane (23.2% of total VOCs), manifesting an intra-source divergence; concurrent concentration increases are observed for iso-butane (+2.3%) and *n*-butane (+2.8%), while decreases occur for *n*-pentane (−17.9%) and iso-pentane (−12.0%). This source contributes substantially (up to 35.2%) to the ozone formation potential (OFP), and its dynamic compositional divergence may undermine emission reduction efficacy. Secondly, structural risks associated with solvent usage sources become prominent, evidenced by persistent emissions of highly reactive species. Ortho-xylene increases by 16.7% and ethylbenzene increases by 40%, whereas propanal decreases by 79.5% and toluene decreases by 14.3% (accuracy 89.2%), reflecting the latent risk of increasing high-reactivity components within substitute solvents. Thirdly, a synergistic effect exists between biogenic and solvent use sources: isoprene is predicted to stabilize at 0.1 ppb (accuracy 71.4%), confirming its characteristic of contributing 10.0% to total OFP despite a low mass fraction (3.9%), while a 14.0% decrease in acetylene signifies the effectiveness of coal combustion clean-up initiatives. These model predictions capture 61.0% of the total VOC composition measured in 2024 (accuracy > 50.0%), thereby furnishing a credible scientific foundation for regional control strategy formulation.

The activity-oriented prioritization (AOP) framework effectively addresses the decoupling challenge between volatile organic compound (VOC) mass concentration and ozone formation potential (OFP) by recalibrating the focus of control strategies—shifting from traditional total VOC reduction to targeted management of highly reactive species. Analysis of Maximum Incremental Reactivity (MIR) coefficients for high/low-concentration reactive VOC components (Figure 12) reveals that deep mitigation of ozone pollution in this region requires prioritizing the following highly reactive VOCs: toluene (4.00 g O_3_/g VOC), ethylbenzene (3.04 g O_3_/g VOC), propanal (7.08 g O_3_/g VOC), o-xylene (7.64 g O_3_/g VOC), and isoprene (10.61 g O_3_/g VOC). Although accounting for a limited proportion of total VOC mass, these species exhibit substantially higher ozone production efficiency per unit mass compared to conventional species (e.g., 1–2 orders of magnitude greater than n-alkanes), with o-xylene and isoprene demonstrating MIR values 27.3 times and 37.9 times that of the benchmark species ethane, respectively. These findings provide a targeted pathway for maximizing OFP reduction efficiency through source-specific abatement of these highly reactive components, grounded in fundamental chemical mechanisms.

## 4. Conclusions

This study establishes a comprehensive understanding of the ongoing ozone pollution dynamics in the Datong region through integrated observational analysis, pollution source apportionment, and predictive modeling. The principal conclusions are summarized as follows:

(1) Photochemical Regime Threshold and Precursor Drivers: A VOC/NO_x_ threshold ratio of 1.5 distinctly delineates the photochemical regimes, characterized as NO_x_-limited in winter and VOC-sensitive in summer. This threshold ratio is robustly validated by the following observations: under high summer ratios, a significant inverse correlation exists between O_3_ and NO_x_ (r = −0.80); whereas during low winter ratios, NO titration predominates (O_3_ + NO → NO_2_). VOC reactivity governs O_3_ production. Despite an overall decline in total VOC concentrations (reduced by 60.1% from peak levels), high-O_3_ events (>160 μg m^−3^) persistently occur when VOC concentrations exceed 17 ppb under low NO_x_ conditions (8–36 μg m^−3^) and elevated NO_2_/NO_x_ ratios (~95%), confirming VOC-sensitive chemistry.

(2) Positive Matrix Factorization (PMF) source apportionment reveals four dominant contributors to VOC pollution in this region: natural gas/fuel gas use and leakage, solvent usage, traffic emissions, and biogenic sources. The natural gas/fuel gas use and leakage source is the predominant contributor to light alkanes (ethane, propane, butanes), accounting for 49.6–66.9% of their total concentration. Traffic emissions are the major source of methyl tert-butyl ether (MTBE, 87.9%) and methylcyclohexane (68.5%). Solvent usage constitutes the primary source for key aromatic hydrocarbons, exemplified by ethylbenzene (71.1%) and o-xylene (71.1%). Biogenic emissions are unambiguously identified as the principal source of isoprene (84.0%).

(3) PMF-OFP coupled analysis reveals three critical source–component linkages: (i) Natural gas/fuel gas use and leakage dominates the total pollutant load (48.3% of VOCs) and contributes 48.3% of total OFP, primarily attributable to propane—projected to increase by 17.1% in 2025—despite its moderate per-mass reactivity. (ii) Solvent usage sources drive 37.1% of the total OFP, primarily through highly reactive aromatics, notably toluene (contributing 17.8% of OFP) and o-xylene/1,2,4-trimethylbenzene (collectively 12.8% of OFP). (iii) Biogenic sources exert a disproportionate influence on the OFP via isoprene emissions (10.0% of OFP vs. 2.6% mass contribution), necessitating regional BVOC mitigation strategies despite their limited mass fraction.

(4) GM (1,1) projections indicate a mounting challenge. Despite a reduction in volatile organic compound (VOC) emissions (−15.7%) and only a marginal increase in nitrogen oxides (NO_x_) emissions (+2.4%), ozone (O_3_) concentrations are predicted to rise to 169.7 μg m^−3^ (+7.4%), signifying enhanced photochemical reaction efficiency. Prioritized abatement targets include aromatic hydrocarbons from solvents, natural gas/fuel gas use and leakage, and regional biogenic volatile organic compounds (BVOCs).

(5) This activity-oriented prioritization (AOP) framework effectively addresses the decoupling challenge between ozone formation potential and emission levels in volatile organic compounds (VOCs) by shifting the regulatory focus from total mass concentration to precise control of highly reactive species. For ozone pollution mitigation in Datong City, implementing tiered control strategies targeting high-reactivity VOCs—specifically toluene, ethylbenzene, propanal, o-xylene, and isoprene—based on their Maximum Incremental Reactivity (MIR) coefficients is imperative.

By transcending conventional mass-based control approaches, this paradigm provides actionable guidance to industrial cities grappling with ozone precursor mitigation challenges as they address temperature variations—particularly seasonal fluctuations. Should the integration of Datong City’s emission inventory with environmental volatile organic compound (VOC) monitoring data be achieved, it would allow for the development of more targeted emission reduction strategies. In turn, this could significantly enhance pollution control effectiveness. Such an integrated approach holds the potential to provide robust scientific evidence supporting the coordinated pollution and carbon management strategies with precision outlined in China’s upcoming 15th Five-Year Plan.

## Data Availability

The data presented in this study are available on request from the corresponding author.
